# Platelet IP6K1 regulates neutrophil extracellular trap–microparticle complex formation in acute pancreatitis

**DOI:** 10.1172/jci.insight.148169

**Published:** 2021-02-22

**Authors:** Raeh Madhi, Milladur Rahman, Dler Taha, Johan Linders, Mohammed Merza, Yongzhi Wang, Matthias Mörgelon, Henrik Thorlacius

Original citation: *JCI Insight*. 2019;e129270. https://doi.org/10.1172/jci.insight.129270

Citation for this retraction: *JCI Insight*. 2021;6(4):e148169. https://doi.org/10.1172/jci.insight.148169

Following a review by the Swedish National Board for Assessment of Research Misconduct, it was determined that a serious breach of good scientific practice in the form of falsification occurred during the pseudocoloring of scanning electron micrographs in this article. The National Board determined that the serious deviation from good research practice was not intentionally committed and concluded that no research misconduct occurred. The paper is being retracted because *JCI Insight* editorial policy prohibits falsification and misrepresentation of data.

## Version 1 02/22/2021

Electronic publication

## Version 2 03/10/2021

The original citation date was corrected.

